# Examination of paraben release from baby teethers through migration tests and GC–MS analysis using a stable isotope dilution assay

**DOI:** 10.1186/s13065-019-0587-6

**Published:** 2019-05-23

**Authors:** Theodoros Potouridis, Alena Knauz, Elisabeth Berger, Wilhelm Püttmann

**Affiliations:** 1 Department of Environmental Analytical Chemistry, Institute for Atmospheric and Environmental Sciences, Goethe-University Frankfurt am Main, 60438 Frankfurt am Main, Germany; 2 Faculty of Biology, Department Aquatic Ecotoxicology, Goethe-University Frankfurt am Main, 60438 Frankfurt am Main, Germany; 3 Department of Quantitative Landscape Ecology, Institute for Environmental Sciences, University Koblenz-Landau, 76829 Landau, Germany

**Keywords:** Parabens, Sorbic acid, Chemical preservatives, Gel-filled baby teether, Ethylene-vinyl acetate (EVA), Migration study, Solid-phase extraction (SPE), Stable isotope dilution assay (SIDA), Gas chromatography–mass spectrometry (GC–MS)

## Abstract

Parabens and sorbic acid are commonly used as food preservatives due to their antimicrobial effect. However, their use in foods for infants and young children is not permitted in the European Union. Previous studies found these compounds in some gel-filled baby teethers, whereby parabens, which are well-known as endocrine disruptors, were identified in the polymer-based chewing surface consisting of ethylene-vinyl acetate (EVA). To assess the exposure of infants and young children to these products, the application of parabens in teethers should be thoroughly investigated. Therefore, the present study aimed to apply a representative migration test procedure combined with an accurate analytical method to examine gel-filled baby teethers without elaborate sample preparation, high costs, and long processing times. Accordingly, solid-phase extraction (SPE), in combination with a stable isotope dilution assay (SIDA) and subsequent gas chromatography–mass spectrometry (GC–MS) for analysis of methyl-, ethyl-, and *n*-propylparaben (MeP, EtP, and *n*-PrP), was found to be well-suited, with recoveries ranging from 93 to 99%. The study compared the release of these parabens from intact teether surfaces into water and saliva simulant under real-life conditions, with total amounts of detected parabens found to be in the range of 101–162 µg 100 mL^−1^ and 57–148 µg 100 mL^−1^, respectively. Furthermore, as a worst-case scenario, the release into water was examined using a long-term migration study.

## Introduction

Consumer goods for infants and young children are examined increasingly rigorously for safety. For example, many studies have shown that phthalic acid esters are present in different types of baby teethers (including water-filled teethers), toys, and other childcare products consisting of polyvinyl chloride (PVC) [1–4]. Phthalic acid esters are commonly used as plasticisers for PVC-based products. However, as phthalate plasticisers are endocrine-disrupting compounds (EDCs) [5], they are considered particularly critical if they can leach out of their polymeric materials, as has been demonstrated [2–4]. Therefore, intake into the body cannot be excluded, particularly for teethers. Consequently, in 2005, the European Parliament and Council restricted the use of phthalic acid esters in toys and childcare products [6–8].

Additives other than plasticisers also present safety risks in toys and childcare products. For example, antimicrobial substances in the gel fillings of baby teethers were recently determined in both the gel fillings and surrounding plastic materials [9, 10]. A study on the endocrine activity of different types of baby teethers detected estrogenic and antiandrogenic effects for one gel-filled product [9]. The causative chemicals identified were alkyl esters of *p*-hydroxybenzoic acid (parabens) found in the polymer-based chewing surface, which consisted of copolymer ethylene-vinyl acetate (EVA). In contrast, parabens were not identified in teethers made of solid plastic or water-filled products [9]. Moreover, the parabens detected were confirmed to migrate from the intact surface of the gel-filled baby teether into water [9]. Similar to phthalate plasticisers, parabens also act as endocrine disruptors [11–13].

A follow-up study that focused on establishing an analytical method for the quantification of methyl-, ethyl-, and *n*-propylparaben (MeP, EtP, and *n*-PrP) reported the methanol-extractable paraben amounts from EVA-based chewing surfaces of the respective gel-filled baby teether products [10]. Furthermore, the gel fillings were examined to verify whether parabens detection in the EVA polymer was directly connected to their presence in the gel material. Results showed that parabens and (2*E*,4*E*)-hexa-2,4-dienoic acid (sorbic acid) were present in all gel fillings [10]. Parabens and sorbic acid are usually used as additives in pharmaceuticals, cosmetics, and food products [14–16] due to their antimicrobial effects [15, 17, 18]. Microbial growth is conceivable in gels due to their high water content. As the total amount of detected parabens in both the EVA polymer and gel material was as high as several hundred µg g^−1^ (0.03–0.12%, w/w material), these results were thought to represent the intentional deployment of parabens and sorbic acid, rather than random product contamination.

The detected levels of parabens were typical of their application as preservatives, such as in cosmetic products. The European Regulation (EC) No 1223/2009 [19, 20] on cosmetic products permits the addition of MeP and EtP and their salts at a maximum concentration of 0.4% per individual ester, and *n*-PrP and *n*-butylparaben (*n*-BuP) and their salts at a maximum concentration of 0.14% for the sum of individual esters (w/w, calculated as acid). For mixtures, a maximum total concentration of 0.8% (w/w, calculated as acid) is permitted if the sum of *n*-PrP and *n*-BuP and their salts does not exceed 0.14%. MeP and EtP and their sodium salts (E 214–219) and sorbic acid and its salts (E 200–203) are also permitted additives in the EU for food preservation, with their use restricted by European Regulation (EU) No 1333/2008 [21, 22]. However, the use of parabens in gel-filled baby teethers should receive special attention because these compounds are not permitted for the preservation of foods for infants and young children in the EU. Therefore, the present study focuses on conducting a representative migration study to obtain quantitative data of parabens leachability from gel-filled baby teethers with EVA-based chewing surfaces under realistic conditions for risk assessment. Furthermore, the aim was to establish an accurate and reliable method for parabens quantification from migration test solutions using a simple instrumental set-up combined with facile analyte extraction.

If the analysis of many samples is required, solid-phase extraction (SPE) provides faster sample processing than other extraction techniques, including LLE in a separatory funnel. Another advantage of SPE is the low amount of solvent used, which results in faster processing post-extraction, such as solvent removal by rotary evaporator. Moreover, SPE generally allows different types of stationary phase to be used in combination with appropriate procedures, such as the choice of elution solvent, for analyte extraction from a wide range of possible sample matrices. This might be useful to establish a selective sample preparation method for known compounds. In particular, when compounds extracted from complex matrices are insufficiently separated by chromatography, resulting in the overlap of qualifier and quantifier ions of co-eluting compounds during mass spectrometry, such a sample preparation step is essential to obtain reliable quantitative data.

Therefore, quantitative data were obtained in the present study using SPE with subsequent extract analysis by gas chromatography–mass spectrometry (GC–MS) on *N*,*O*-bis(trimethylsilyl)trifluoroacetamide (BSTFA)-derivatised parabens in combination with a stable isotope dilution assay (SIDA).

Furthermore, the detected paraben concentrations released from gel-filled baby teethers into water and saliva simulant were discussed in connection with the use of EVA polymer as a chewing surface material and compared with maximum values for food preservation established by the EU.

## Experimental

### Materials and chemicals

Methyl 4-hydroxybenzoate (methylparaben, MeP; purity ≥ 99%), ethyl 4-hydroxybenzoate (ethylparaben, EtP; purity ≥ 99%), *n*-propyl 4-hydroxybenzoate (*n*-propylparaben, *n*-PrP; purity ≥ 99%), potassium (2*E*,4*E*)-hexa-2,4-dienoic acid (potassium sorbate; purity ≥ 99%), and sodium sulfate (purity ≥ 99%) were obtained from Aldrich (Steinheim, Germany). Methyl 4-hydroxybenzoate-2,3,5,6-[^2^H_4_] (methylparaben-*d*_*4*_, MeP-*d*_*4*_; purity 99%, isotopic enrichment 98 atom% D) was purchased from CDN Isotopes (Quebec, Canada). Methanol (purity ≥ 99.9%), dichloromethane (purity ≥ 99.5%), acetone (purity ≥ 99.9%), and 37% hydrochloric acid (HCl, extra pure) were obtained from Carl Roth (Karlsruhe, Germany). Methanol and dichloromethane were distilled before use. Silylation reagent *N*,*O*-bis(trimethylsilyl)trifluoroacetamide (BSTFA) was purchased from CS-Chromatographie (Langerwehe, Germany). Calcium chloride (purity ≥ 97%) was obtained from AppliChem (Darmstadt, Germany). Magnesium chloride hexahydrate (purity ≥ 99%) was purchased from neoLab Migge (Heidelberg, Germany). Sodium chloride (purity ≥ 99.5%) and dipotassium hydrogen phosphate (purity ≥ 99%) were obtained from VWR International (Darmstadt, Germany). Potassium carbonate (purity ≥ 99%), potassium chloride (purity ≥ 99.5%), and pyridine (purity ≥ 99.5%) were purchased from Merck Chemicals (Darmstadt, Germany). Ultrapure water (deionised) was prepared using an Astacus Analytical system from membraPure (Bodenheim, Germany) using a TwinPak cartridge (with organic scavenger) from Purefekt (Karlsruhe, Germany).

### Samples

Gel-filled baby teethers from three different brands were purchased from local and internet retailers in Germany in 2015. The three brands considered were from three internationally operating companies selling their products worldwide. The products were labelled as manufactured in China and Thailand. All teethers were labelled as ‘BPA free’ (containing no bisphenol A), while two were additionally labelled as ‘phthalate free’. All teethers were closed rings, but the product designs varied. Only one teether was pacifier-shaped. All teether products were stored in their original packaging at room temperature (23 ± 2 °C) until examination. In total, nine different teethers were analysed by suspected-target and target screening, as previously described [10]. To examine the release behaviour of parabens from the teethers into water and saliva simulant, four teethers containing lower and higher paraben amounts in the polymer-based chewing surface were selected. Furthermore, one of these four teethers (no. 2) was selected to examine paraben release into water in a long-term migration study. The teether selected for this part of the study showed the highest paraben release in the conducted migration tests.

For each teether design, several products with the same lot number were purchased so that the different migration test results were comparable. Teethers were analysed in duplicate (n = 2) in each test. For baby teether no. 2, the product used for the suspected-target and target screening of parabens in the polymer-based chewing surface and gel material had the same lot number as teethers used in the migration tests.

The polymeric material of all teether chewing surfaces consisted of EVA, as determined by Fourier transform infrared (FTIR) spectroscopy using a method described previously [9]. The thickness of the EVA-based outer layer of each product ranged from approximately 1 to 2 mm. The vinyl acetate (VA) content of EVA polymer and the composition of the gel fillings were unknown.

### Material analysis

Quantitative analysis of parabens and the identification of sorbic acid in the EVA-based chewing surface and gel material were conducted as previously described [10]. In brief, gel fillings were separated from the chewing sections and 1 g of each component was subjected to ultrasound-assisted extraction (UAE) for 1 h using methanol as solvent. Aliquots of the methanolic solutions were then processed to perform analyses on BSTFA-derivatised compounds prior to GC–MS analysis. Results were obtained from single analyses.

As previously discussed [10], the UAE method applied to the analysis of MeP, EtP, and *n*-PrP in EVA polymer might not be sufficient to achieve total extraction. However, this was important because internal standard MeP-*d*_*4*_ used for quantification could not be introduced homogenously into the plastic polymer without possible negative effects on the original sample, such as loss of the target compounds. Therefore, possible incomplete extraction could not be compensated for. To examine the extent of the applied extraction method, the chewing section of baby teethers no. 1, 2, 3, and 4 (1 g each) were extracted by UAE four times consecutively for 1 h using fresh methanol for each extraction.

### Migration test

The gel-filled baby teethers were first washed with warm soapy water according to manufacturer instructions. As soap can contain parabens, a paraben-free product was used (liquid soap purchased from a local store in Frankfurt am Main, Germany). The suitability of the soap was tested during method validation on chewing section pieces of a teether that had not shown parabens contents in the material analysis. The teethers were stored at room temperature for at least 12 h to simulate usual conditions of use after cleaning and before cooling for the next use. During storage, parabens from inside the teether might migrate to the surface. Immediately before examination, the teethers were cooled in a domestic refrigerator for 40 min.

The migration test with water was conducted using ultrapure water at pH 6.9. For the saliva simulant, a stock solution containing different salts was prepared in ultrapure water at pH 6.8 (adjusted with 3 mol L^−1^ HCl) as described elsewhere [23]. The salt concentrations used in ultrapure water were as follows: dipotassium hydrogen phosphate, 0.57 g L^−1^; potassium carbonate, 0.53 g L^−1^; sodium chloride, 0.33 g L^−1^; potassium chloride, 0.75 g L^−1^; magnesium chloride 0.08 g L^−1^; and calcium chloride, 0.11 g L^−1^. Both migration tests were conducted under the same conditions, as follows. The teethers were placed in crystallising dishes (borosilicate glass type 3.3 dishes; volume, 300 mL; diameter, 95 mm; height, 55 mm) so that approximately 10 cm^2^ (± 1 cm^2^) of the EVA-based chewing surface was covered with migration test solution (100 mL). The contact surface was randomly selected from the entire chewing surface for each teether product. Following a contact time of 1 h at room temperature (23 ± 2 °C) on an orbital shaker (KS 15 A, Edmund Bühler, Hechingen, Germany) at 100 rounds min^−1^, the solutions were transferred into glass Erlenmeyer flasks and spiked with internal standard prior to analyte extraction. An isotope-labelled standard was added to conduct parabens quantification using a SIDA [24–26]. MeP-*d*_*4*_ was used at a concentration of 59 µg 100 mL^−1^ in the migration test solution (24 µL of 2.46 mg mL^−1^ MeP-*d*_*4*_ standard solution in methanol).

To examine the continuous release behaviour of parabens migrating from 10 cm^2^ (randomly selected) of the EVA-based chewing surface, one teether (no. 2) was subjected to an additional long-term migration study using water under the same test conditions described above. After 1 h, and then on a daily basis, the test solution was replaced with fresh ultrapure water until the paraben concentrations measured in the solution plateaued.

### Solid-phase extraction

Parabens extraction from the water and saliva simulant migration test solutions was performed using octadecyl-bonded silica gel SPE cartridges (endcapped, 500 mg of sorbent, 3-mL cartridge, Supelclean ENVI-18, Supelco, Bellefonte, USA; purchased from Aldrich, Steinheim, Germany). A Vac Elut 24 station (Varian, Darmstadt, Germany) was used as the SPE vacuum manifold. The extraction procedure included a pH adjustment for both test solutions to 4 ± 0.5 (water and saliva simulant adjusted using 0.02 and 1 mol L^−1^ HCl, respectively). This pH level has been reported as optimal in combination with LiChrolut EN sorbent for parabens extraction from environmental water samples, because parabens are present in neutral form [27]. Based on reversed-phase conditions, analytes in neutral form should generally be extracted more efficiently from aqueous solutions when using sorbents with a hydrophobic character. The SPE cartridges were conditioned before use with acetone (5 mL), methanol (5 mL), and ultrapure water (5 mL) at pH 4 (adjusted with 0.02 mol L^−1^ HCl). The pH-adjusted samples were then passed through the cartridges at a flow rate of approximately 2 mL min^−1^ using Teflon tubing with adapters. Subsequently, the sorbent material was dried under nitrogen flow (15 min at a pressure of 2 bar inside the SPE cartridge; gas supplied by Praxair, Düsseldorf, Germany; gas purity 5.0) and the parabens were then eluted into glass vials using methanol (2 mL).

Prior to GC–MS analysis, 50 µL of each methanol extract was taken to convert the parabens into trimethylsilylesters with BSTFA as previously described [10]. Subsequently, the derivatised parabens were analysed by GC–MS with automated sample injection.

### GC–MS operating conditions

Paraben analysis was conducted by GC–MS as previously described for the material analysis of similar gel-filled baby teether products [10]. In brief, a Trace GC Ultra oven (ThermoFisher Scientific, Dreieich, Germany) was coupled to a DSQ II detection system (quadrupole MS, ThermoFisher Scientific, Dreieich, Germany) and equipped with a 30 m × 0.25 mm i.d. fused silica capillary coated with 5% diphenyl/95% dimethylpolysiloxane at a film thickness of 0.25 µm (ZB-5MSi, Phenomenex, Aschaffenburg, Germany). A constant carrier gas flow of 1.1 mL helium min^−1^ was used (gas supplied by Praxair, Düsseldorf, Germany; gas purity 5.0). The GC inlet port temperature was set to 240 °C and the oven temperature programme was initiated at 40 °C (1 min isothermal for suspected-target screening; 2 min isothermal for target screening), then increased to 130 °C at 25 °C min^−1^, to 260 °C at 4 °C min^−1^, and finally to 300 °C at 25 °C min^−1^ (15 min hold time). The transfer line coupling the GC oven to the MS detector was set to 280 °C. Mass detection was performed on positive ions after electron ionisation (EI+) at 70 eV. The ion source temperature was set to 220 °C.

To identify the target compounds for suspected-target screening, derivatised sample extracts were analysed undiluted in full scan mode (*m/z* 50–650, scan rate of 2 scans s^−1^) by injecting 1 µL of each dichloromethane solution in splitless mode. For quantification (target screening), derivatised sample extracts were analysed undiluted or after dilution with methanol (prior to derivatisation) in selected ion monitoring mode by injecting 2 µL of each dichloromethane solution in split mode (split flow of 11 mL min^−1^). The qualifier and quantifier ions (in bold) selected for mass detection were: *m/z* = 193 (197), 209 (213), **224** (**228**) for MeP (MeP-*d*_*4*_); *m/z* = **193**, 223, 238 for EtP; and *m/z* = **193**, 237, 252 for *n*-PrP. The quantifier ion for MeP is the molecular ion of the mass spectrum and the fragment ions selected for quantification of EtP and *n*-PrP are the base peaks of the respective mass spectra. The mass scan width was *m/z* = 1 for all masses and the dwell time was 100 ms.

Data acquisition, instrumental operation, and data analysis were conducted using Xcalibur software (version 2.0.7, ThermoFisher Scientific, Dreieich, Germany). NIST MS Search software (version 2.0, National Institute of Standards and Technology, Gaithersburg, Maryland, USA) was used to perform mass spectra searches.

### Identification of parabens and sorbic acid

BSTFA derivatives of MeP, EtP, *n*-PrP, and sorbic acid in samples for suspected-target screening were identified as previously described [10]. The mass spectra obtained and linear retention indices (LRIs) [28, 29] determined using the slightly polar ZB-5MSi GC separation column were compared to those of derivatised reference substances.

### Method calibration

The quantification of MeP, EtP, and *n*-PrP in samples of EVA polymer and gel materials from baby teethers was based on linear calibration curves in the concentration range 10–200 µg 10 mL^−1^ methanol, as previously described [10].

To quantify parabens release from the EVA-based chewing surface into water and saliva simulant in the migration tests, seven concentration levels ranging from 10 to 150 µg 100 mL^−1^ migration test solution (10, 25, 50, 75, 100, 125, and 150 µg 100 mL^−1^; n = 1 each) were prepared for MeP, EtP, and *n*-PrP. The single calibration solutions of both sets contained a constant concentration level of MeP-*d*_*4*_ (59 µg 100 mL^−1^) as internal standard. The resulting concentration ratios between each paraben and the internal standard were approximately 0.2–2.5. For calibration graphs, the area ratios obtained were plotted against concentrations (y, paraben/MeP-*d*_*4*_ area ratio; x, paraben concentration). Both calibration sets (water and saliva simulant) were treated in the same way as the real samples by applying the shaking procedure in the crystallising dish prior to adding MeP-*d*_*4*_ as described above. Based on the calibration data obtained, the limits of detection (LOD) and quantification (LOQ) for MeP, EtP, and *n*-PrP were estimated using a mathematical/statistical approach according to German standard DIN 32645 [30].

### Method validation

The repeatability of the SPE/GC–MS method applied to the analysis of parabens from water and saliva simulant was evaluated for each migration solution matrix in a set of five independently spiked test solutions. For each migration solution, 100 mL was prepared containing a defined amount of approximately 75 µg of MeP, EtP, and *n*-PrP. These solutions were also used to evaluate the recovery efficiency of the applied SPE/GC–MS method.

To control contamination from the laboratory equipment and environment, procedural blanks of water and saliva simulant were used for each baby teether sample batch (n = 2 each).

The test solution sets prepared in water and saliva simulant, and the procedural blanks, were treated in the same way as the real samples by applying the shaking procedure in the crystallising dish prior to adding MeP-*d*_*4*_ as described above.

The suitability of the purchased paraben-free soap product, used to prepare the warm soapy water solution to wash the baby teethers before performing migration tests, was examined as follows: pieces of the chewing section (10 cm^2^) from a teether that had not shown any parabens by material analysis were prepared and washed as described above. Two pieces were then subjected to the migration test with water and saliva simulant.

## Results and discussion

### Identification of parabens and sorbic acid

**Fig. 1:**
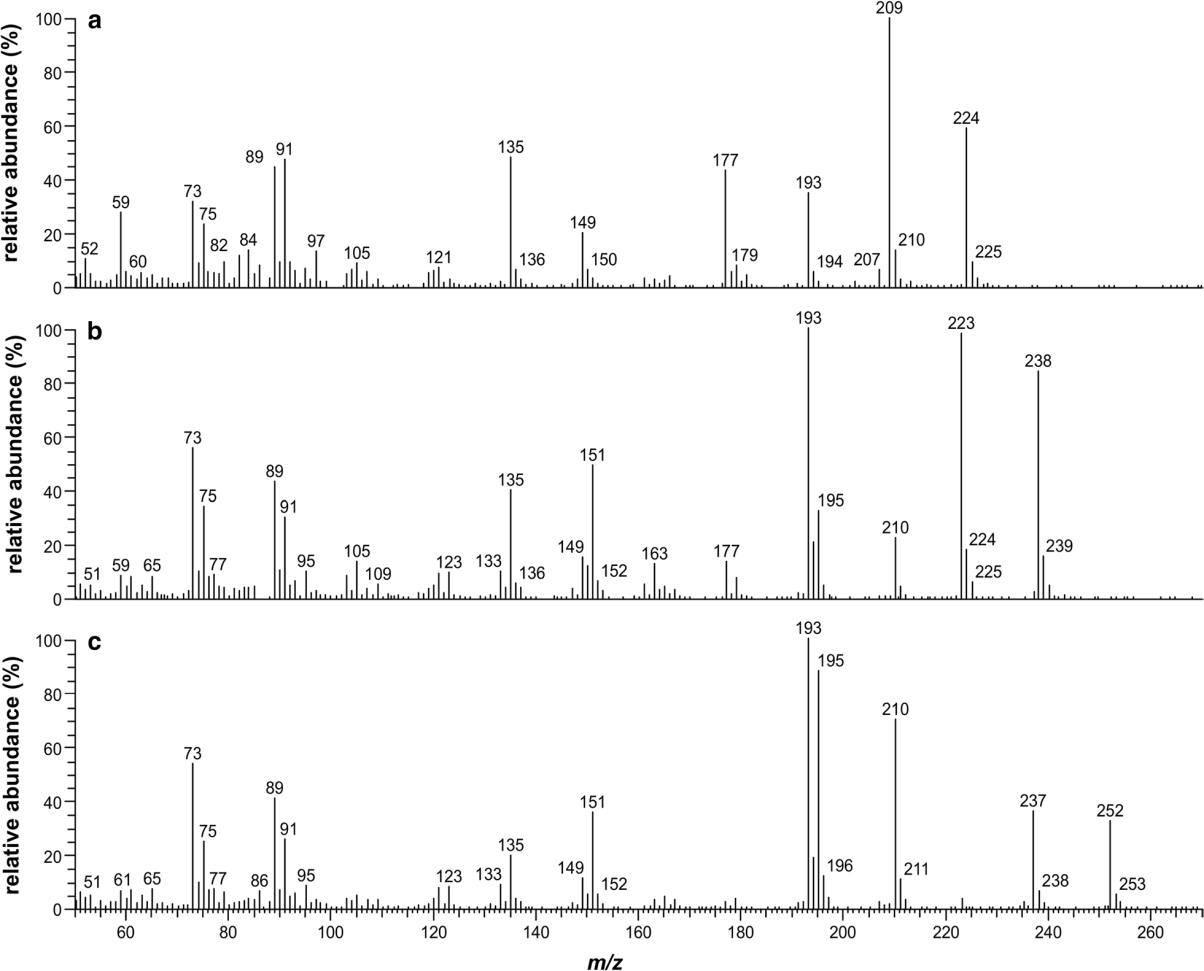
Mass spectra of trimethylsilylated **a** methylparaben, **b** ethylparaben, and **c** *n*-propylparaben. Positive electron ionisation mode (EI+, 70 eV); quadrupole mass spectrometer

**Fig. 2:**
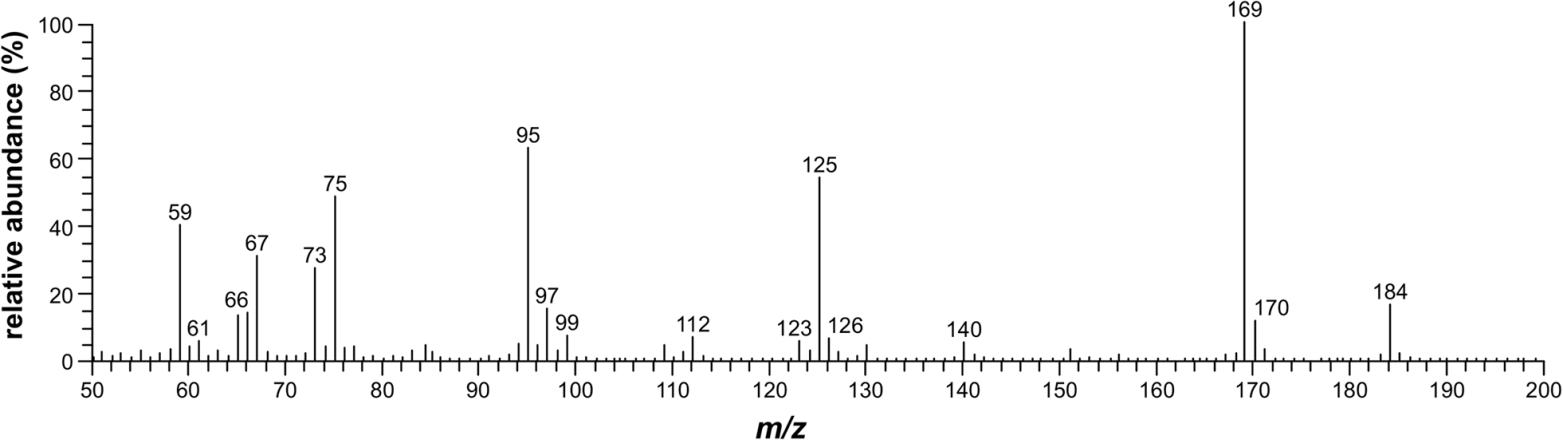
Mass spectrum of trimethylsilylated (2*E*,4*E*)-hexa-2,4-dienoic acid (sorbic acid). Positive electron ionisation mode (EI+, 70 eV); quadrupole mass spectrometer

The mass spectra and determined LRI values of BSTFA derivatives of MeP, EtP, *n*-PrP, and sorbic acid detected in the EVA polymer and gel material, and in the corresponding water and saliva simulant migration test solutions, showed good agreement with those of derivatised reference substances. Match factors of ≥ 850 (direct match) with available reference mass spectra from the NIST library were achieved. For silylated EtP, no mass spectrum was available in the NIST library. Mass spectra of trimethylsilylesters of MeP, EtP, *n*-PrP, and sorbic acid are shown in Figs. 1 and 2. The LRIs determined on ZB-5 were 1494 (1495) for trimethylsilylated MeP (MeP-*d*_*4*_), 1568 for trimethylsilylated EtP, 1667 for trimethylsilylated *n*-PrP, and 1183 for trimethylsilylated sorbic acid. The LRI value of derivatised MeP was in good agreement with available NIST data (reference LRI value, 1504). For trimethylsilylesters of EtP, *n*-PrP, and sorbic acid, no LRI values were available in the NIST library.

Paraben preservatives were detected in seven of the nine teethers examined in this study. Among these, five teethers showed combined use of MeP and *n*-PrP, while two teethers contained MeP and EtP. Furthermore, analyses of the gel of five teethers from the same brand showed the combination of parabens with sorbic acid. These findings were in agreement with previously obtained results for similar gel-filled baby teether products [10]. However, two teethers from the same brand showed no detectable parabens, indicating that the respective manufacturer did not use parabens and sorbic acid in their products.

### Method calibration

For MeP, EtP, and *n*-PrP, good linearities were achieved in the concentration range 25–125 µg 100 mL^−1^ (concentrations of 10 µg and 150 µg 100 mL^−1^ were excluded as outliers) in both water and saliva simulant, with *R*^2^ values of 0.999 for each paraben. This specified calibration range corresponded to an absolute amount of each paraben of approximately 0.57–2.87 ng on the GC separation column (calculated by considering the applied split flow during sample injection, but not considering, for example, possible analyte losses during SPE). The difference between theoretical and calculated concentrations for each calibration level was less than 8.5%. Calibration curve data obtained for MeP, EtP, and *n*-PrP, and their estimated LOD and LOQ values, are summarised in Table 1.

**Table 1:** Calibration curve data for methylparaben (MeP), ethylparaben (EtP), and *n*-propylparaben (*n*-PrP) in water and saliva simulant

Compound	*R* ^2^	LOD (µg 100 mL^−1^)	LOQ (µg 100 mL^−1^)
Water			
MeP	0.9989	7.1	25.3
EtP	0.9992	6.2	22.2
*n*-PrP	0.9986	7.9	27.5
Saliva simulant			
MeP	0.9992	6.1	21.8
EtP	0.9993	5.6	20.2
*n*-PrP	0.9987	7.7	27.1

Five calibration points each; n = 1; calibration range, 25–125 µg 100 mL^−1^

*LOD* limit of detection, *LOQ* limit of quantification

### Method validation

In both water and saliva simulant procedural blank, MeP, EtP, *n*-PrP, other parabens, and sorbic acid were not detected. Therefore, there was no risk of cross-contaminating real samples. The paraben-free soap used to wash the baby teethers also showed no parabens content, making it suitable for the migration study.

Good recoveries of 93, 98, and 97% were obtained for MeP, EtP, and *n*-PrP in water, and of 99, 94 and 95%, respectively, in saliva simulant (Table 2). Repeatability measurements of MeP, EtP, and *n*-PrP in both water and saliva simulant showed good analytical precision, with relative standard deviations (RSDs) lower than 4.5% (Table 2).

**Table 2:** Recovery and repeatability data for analysis of methylparaben (MeP), ethylparaben (EtP), and *n*-propylparaben (*n*-PrP) from spiked water and saliva simulant

Compound	Spiking concentration (µg 100 mL^−1^)	Recovery (mean ± SD, %)	Repeatability (RSD, %)
Water			
MeP	76	93 ± 3.9	4.2
EtP	75	98 ± 3.1	3.2
*n*-PrP	75	97 ± 2.4	2.4
Saliva simulant			
MeP	76	99 ± 1.0	1.0
EtP	75	94 ± 0.5	0.5
*n*-PrP	75	95 ± 1.0	1.1

Repeatability expressed as relative standard deviation (RSD). Calculated from five independently spiked solutions each

### Paraben concentrations in gel-filled baby teethers

The analytical results, as summarised in Table 3, show the amounts of parabens found in the EVA polymer and gel material of the gel-filled baby teethers selected for migration studies. For the seven paraben-positive teethers from two different brands, the total parabens contents ranged from 283 to 1242 µg g^−1^ in the EVA polymer and from 164 to 593 µg g^−1^ in the gel material (for individual results, see Table 3; results for teethers not selected for migration studies are not shown).

**Table 3:** Methylparaben (MeP), ethylparaben (EtP), and *n*-propylparaben (*n*-PrP) release from gel-filled baby teethers into water and saliva simulant and their detected concentrations in plastic and gel material

Baby teether	Migration test/product material	Individual paraben concentrations			Total paraben amount	Units
		MeP	EtP	*n*-PrP		
No. 1	Water	41 (17.3)	< LOD	62 (31.6)	103	µg 100 mL^−1^
No. 1	Saliva simulant	34 (7.3)	< LOD	74 (1.0)	108	µg 100 mL^−1^
No. 1	Plastic	506	< LOD	736	1242	µg g^−1^
No. 1	Gel	367	< LOD	52	419	µg g^−1^
No. 2	Water	54 (8.9)	< LOD	95 (25.3)	149	µg 100 mL^−1^
No. 2	Saliva simulant	46 (3.1)	< LOD	102 (23.2)	148	µg 100 mL^−1^
No. 2	Plastic	258	< LOD	376	634	µg g^−1^
No. 2	Gel	140	< LOD	24	164	µg g^−1^
No. 3	Water	69 (20.6)	< LOD	32 (3.7)	101	µg 100 mL^−1^
No. 3	Saliva simulant	30 (1.8)	< LOD	27 (0.6)^a^	57	µg 100 mL^−1^
No. 3	Plastic	269	< LOD	95	364	µg g^−1^
No. 3	Gel	399	< LOD	23	422	µg g^−1^
No. 4	Water	111 (12.8)	< LOD	51 (1.4)	162	µg 100 mL^−1^
No. 4	Saliva simulant	87 (2.3)	< LOD	47 (6.4)	134	µg 100 mL^−1^
No. 4	Plastic	341	171	< LOD	512	µg g^−1^
No. 4	Gel	519	74	< LOD	593	µg g^−1^

Migration test values: mean concentration with range (number in brackets) from two independently examined products

Product material values: results from single analyses (n = 1)

*LOD* limit of detection, *LOQ* limit of quantification

^a^Mean concentration calculated as 27.2 µg 100 mL^−1^, above the estimated LOQ value of 27.1 µg 100 mL^−1^

Notably, analysis of the chewing section (1 g) in this study comprised paraben amounts extracted from the outer and inner sides of the EVA polymer. Therefore, it should be assumed that higher paraben amounts were detected than would be extracted from 1 g of plastic from an intact chewing surface. Furthermore, parabens might migrate to the inner side of the polymer due to influx of the gel by swelling, which could also have influenced the results.

Besides this, verification of the extraction efficiency of the applied UAE method for analysis of MeP, EtP, and *n*-PrP in the EVA polymer showed that total extraction was not achieved. The results for all four teethers examined showed that successive extracts of the respective EVA sample also contained parabens. The MeP, EtP, and *n*-PrP concentration levels detected from the second extraction were similar to those from the first extraction, whereas the results of the third and fourth extractions were approximately 50% lower than those of the first extraction (data not shown). Therefore, the results obtained from EVA material analysis cannot be considered to reflect the total amount of parabens present in the samples. Solvent extraction using a Soxhlet extractor could be a possible approach to total parabens extraction from the EVA-based chewing section of the gel-filled baby teethers [31–33].

All teethers in which parabens were detected had a particularly strong smell immediately after opening their packaging. The odour was identical and similar in intensity to that of pure reference materials MeP and EtP.

### Paraben release from gel-filled baby teethers into water and saliva simulant

A representative in vitro migration study for analysing chemicals released from baby teethers to obtain reliable data representative of real-life use should consider several critical prerequisites. The test procedures applied should comprise an adequate migration test solution in conjunction with an adjusted temperature, volume, contact time, and compression force to simulate mouthing and chewing conditions during teether use. The present examination was mainly focused on testing paraben release from a surface area representative of that mouthed by children with a corresponding test solution volume and contact time. The defined reference contact surface area of 10 cm^2^ is a typical area to simulate mouthing by a child [3] and test solution volume of 100 mL corresponded to the mean rate of saliva production by active mouthing of an object by an adult over 1 h [3]. Owing to a lack of appropriate equipment, the migration tests were conducted at room temperature (23 ± 2 °C) instead of the optimum temperature of 37 °C (body temperature) and without compression force to simulate chewing conditions [2]. As an alternative to the latter, mechanical agitation was applied using an orbital shaker to simulate saliva movement during normal chewing and sucking of an object. The inorganic salt composition and pH level used for the saliva simulant in this study represented natural saliva compositions as mentioned in literature [23]. The migration test results for the release of MeP, EtP, and *n*-PrP from gel-filled baby teethers into water and saliva simulant obtained under these test conditions are summarised in Table 3.

**Fig. 3:**
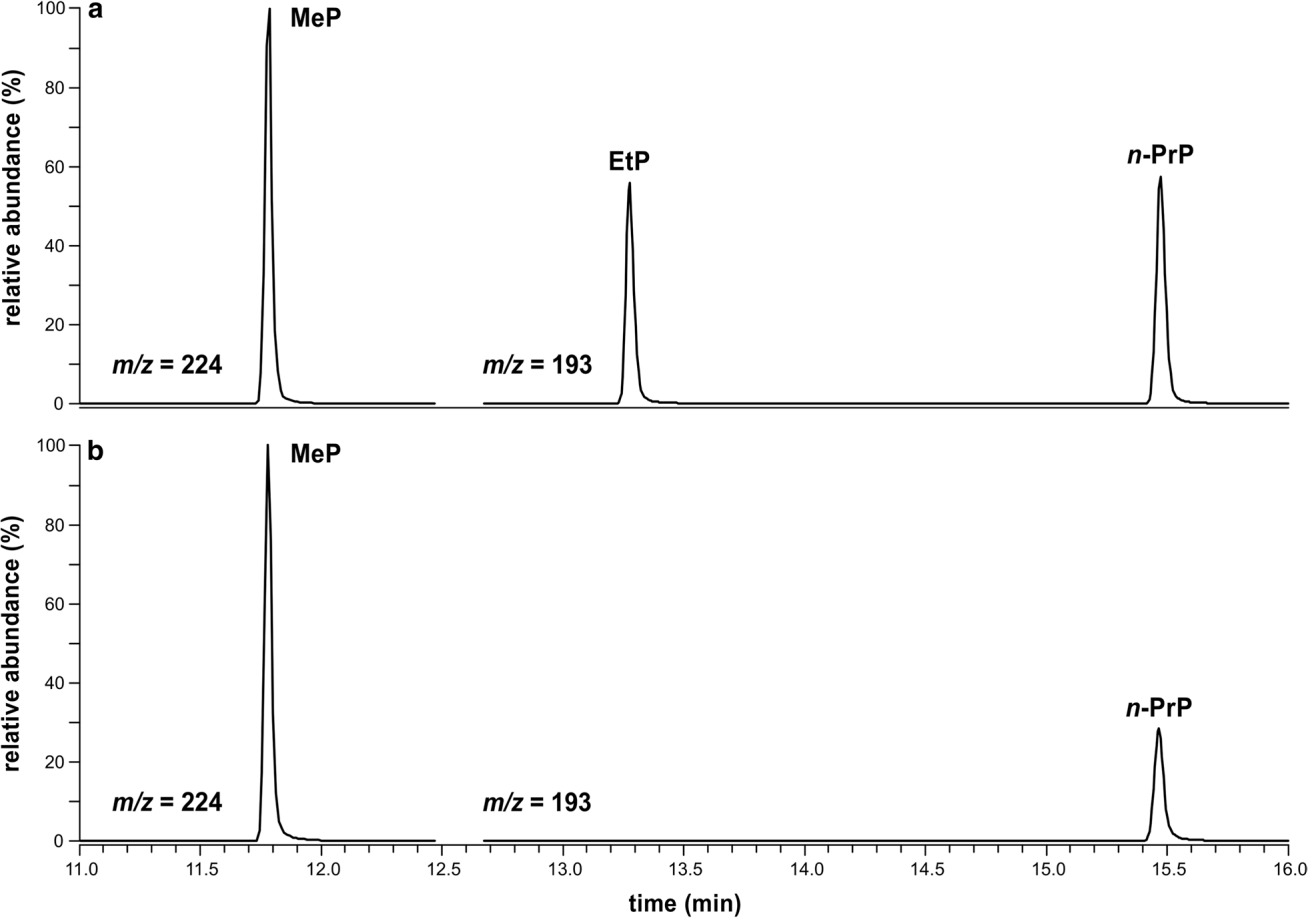
GC–MS chromatograms (target screening) of saliva simulant migration test solutions of **a** calibration level 75 µg 100 mL^−1^ and **b** baby teether no. 4. Extracted ion traces of paraben quantifier ions *m/z* 224 for trimethylsilylated methylparaben (MeP) and *m/z* 193 for trimethylsilylated ethylparaben (EtP) as well as trimethylsilylated *n*-propylparaben (*n*-PrP)

The results confirmed those of a previous study [9] indicating that parabens can migrate from an intact EVA-based chewing surface of a gel-filled baby teether into water. The total amount of parabens released into water and saliva simulant ranged from 101 to 162 µg 100 mL^−1^ and 57 to 148 µg 100 mL^−1^, respectively. Accordingly, major differences between the total paraben amount in water and saliva simulant were not detected. Application of a higher test temperature or a compression force to simulate mouthing and chewing conditions might have led to higher paraben releases from the teethers, also since the solubility of parabens in water increases with temperature [34]. Moreover, depending on the age of the packaged products and their storage conditions, a possible loss of parabens by migration into the packaging material could result in the underestimation of paraben concentrations released into both water and saliva simulant. Exemplary GC–MS chromatograms showing the ion traces of the paraben quantifier ions (target screening) of saliva simulant migration test solutions of calibration level 75 µg 100 mL^−1^ and baby teether no. 4 are illustrated in Fig. 3.

A recent migration study analysed several EDCs from different types of baby teethers [35]. The study reported the presence of parabens in similar gel-filled baby teethers to those examined in the present study, in addition to solid plastic and water-filled teethers. These quantities ranged from 2.0 to 1990 ng teether^−1^ for parent parabens and 0.47 to 839 ng teether^−1^ for their transformation products, with all considered products containing parabens. For parabens as well as their transformation products, the amounts leached from the entire intact surface of the respective teethers into water and subsequently extracted from the water-leached teethers using methanol were summarised. As parent parabens, MeP, EtP, PrP, butyl-, benzyl-, and heptylparaben were detected, as well as the transformation products 4-hydroxybenzoic acid, 3,4-dihydroxybenzoic acid, 3,4-dihydroxybenzoic acid methyl ester, and 3,4-dihydroxybenzoic acid ethyl ester. The applied sample preparation method consisted of a simplified migration test in water with subsequent liquid–liquid extraction (LLE) that required elaborate preparation steps, large amounts of material, and long experiment times. Furthermore, migration tests using saliva simulant were not included, which would have been necessary to confirm the results obtained from migration tests using water and examine whether the detected EDCs, such as parabens, have different leaching behaviours in the presence of salts at pH 6.8. Furthermore, the gel and water fillings themselves were not analysed for possible paraben content and information on the polymer-based chewing surfaces, such as total surface area and particular composition, were not provided. The latter would be important, as the composition of a plastic material plays a major role in its diffusion properties. In particular, organic compounds, as observed for parabens, might migrate into saliva from a product that is supposedly safe for infants and young children. Therefore, the results of the mentioned study, ranging from 2.0 to 1990 ng teether^−1^ [35], cannot be compared with the considerably higher total paraben contents found in the present study, which ranged from 79 to 149 µg 10 cm^−2^ (sum of MeP and *n*-PrP, mean of tests with water and saliva simulant), released from gel-filled baby teethers with EVA-based chewing surfaces.

In both migration tests (water and saliva simulant) on baby teether no. 4, EtP was not detected, despite EtP being present in the directly analysed EVA polymer and gel. Instead of EtP, the teether leached *n*-PrP into the migration test solutions. However, material analysis and migration tests were conducted on teethers of the same design and brand, but with different lot numbers. Therefore, material analysis was repeated with the teether products examined in the migration tests, which showed that EtP had been replaced with *n*-PrP. Obviously, the manufacturer of teethers no. 4 had changed the paraben composition. Furthermore, as no other product contained EtP, no migration results were obtained for EtP in the present study.

### Long-term migration study

**Fig. 4:**
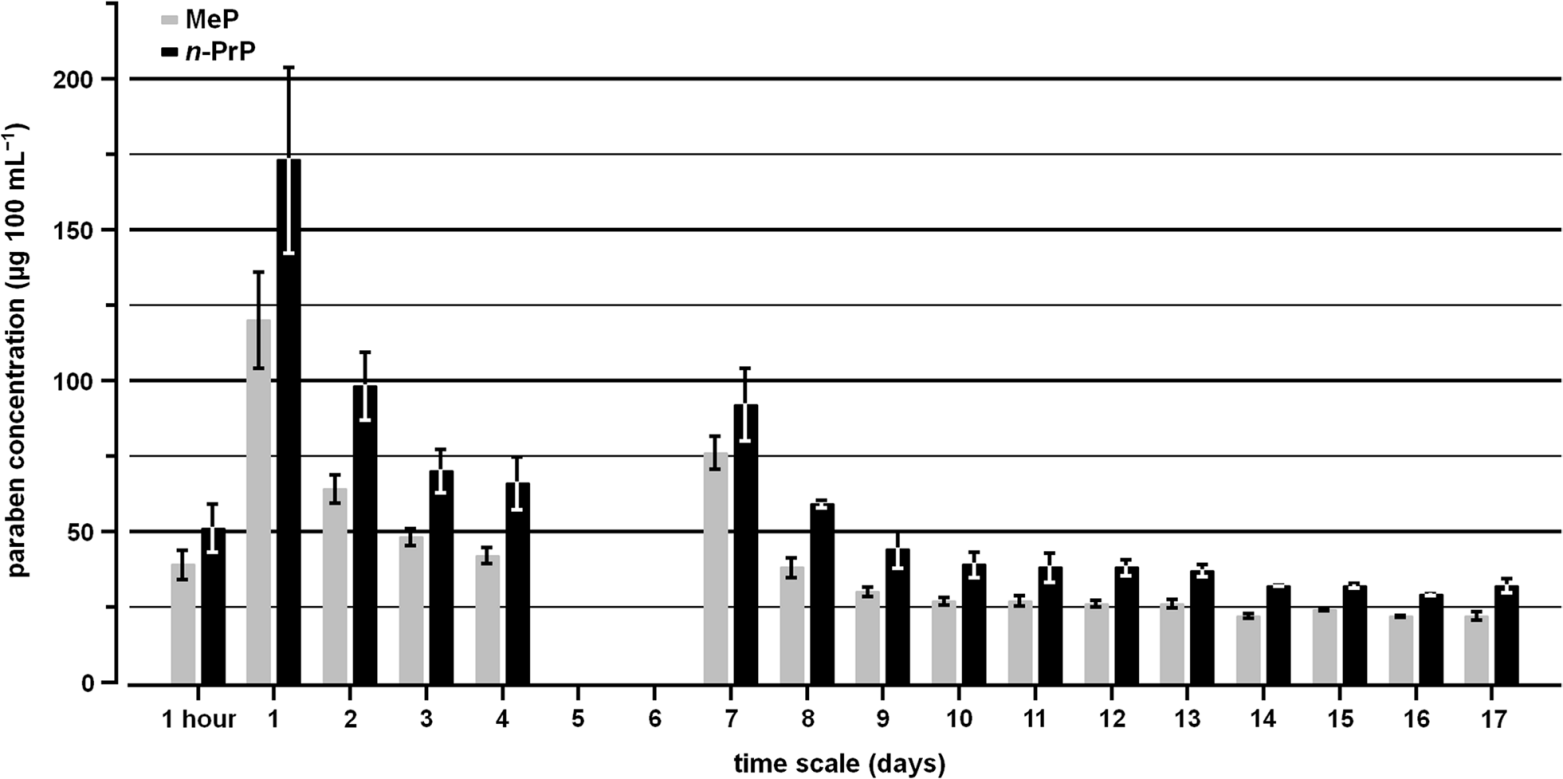
Release behaviour of methylparaben (MeP) and *n*-propylparaben (*n*-PrP) from a gel-filled baby teether into water monitored over a time period of 17 days. Mean concentration values shown with ranges from two independently examined products of the same lot. Sample analyses on day 5 and 6 were not possible. As the MeP mean concentrations on days 14–17 were above the estimated LOD of 7.1 and slightly below the estimated LOQ of 25.3 µg 100 mL^−1^, they were included in this study

No major differences were detected between the total paraben amount released in water and saliva simulant. Therefore, the continuous release behaviour of parabens from the EVA-based chewing surface was monitored using water as the migration test solution. Teether no. 2 was selected for this purpose because it had shown the highest amount of released parabens, accounting for the results of water and saliva simulant analysis. The results of the long-term migration test are shown in Fig. 4. As might have been expected, the initially detected paraben levels of 39 µg and 51 µg 100 mL^−1^ for MeP and *n*-PrP, respectively, had significantly increased by approximately a factor of three after 24 h (120 and 173 µg 100 mL^−1^, respectively). Over the next 3 days, the concentrations of both parabens decreased nonlinearly, showing a possibly exponentially decreasing trend. After 4 days, the concentrations were approximately the same as the initial values of the solution analysed after 1 h. On the 5th and 6th days, daily testing could not be continued, and the experiment was left running without changing the water. Therefore, on the 7th day the concentrations of MeP and *n*-PrP (76 and 92 µg 100 mL^−1^, respectively) had increased again by a factor of approximately two compared with the initial values. Thereafter, the values decreased nonlinearly as before, until concentrations of 22 and 32 µg 100 mL^−1^ for MeP and *n*-PrP, respectively, were reached on the 14th day. After that, no significant concentration changes were observed for both parabens. Therefore, the experiment was terminated on the 17th day.

The total amount of MeP and *n*-PrP continuously released from teether no. 2 into water over 17 days was 1.58 mg 10 cm^−2^. As the amount of parabens detected followed a nonlinear release, the theoretical release per hour was not calculated.

### Risk assessment for the exposure of infants and young children to parabens released from gel-filled baby teethers

To estimate the relevance of the measured paraben release from gel-filled baby teethers to the health of infants and young children, limits determined during safety evaluation of parabens as food additives in the EU were considered.

The European Food Safety Authority (EFSA) published in 2004 [36] that 10 mg kg^−1^ bw (bodyweight) was the temporary acceptable daily intake (ADI) of the sum of MeP and EtP and their sodium salts. However, as ADI values are normally related to adults, it can be assumed that the ADI for infants and young children should be lower. As *n*-PrP has a more severe effect on sex hormones and male reproductive organs in juvenile rats, the substance was excluded from this ADI group. The present migration test results showed that the total amounts of MeP and *n*-PrP (mean of detected amount in water and saliva simulant) released from the four teethers considered were considerably below the ADI of 10 mg kg^−1^ bw day^−1^ (see Table 4). This was in agreement with previously described findings for parabens in the material analysis of similar gel-filled baby teether products [10]. One teether manufacturer labelled its product packaging with a recommendation that the teethers are suitable for children from the age of 3 months, whereas others labelled the teethers for children of 4 months. Based on the first suggestion, calculations were performed based on average bodyweights of 5.8 kg for girls and 6.4 kg for boys at the age of 3 months [37–39]. Even the total amount of MeP and *n*-PrP released into water from teether no. 2 within 17 days was well below the ADI value.

**Table 4:** Comparison of total paraben amount released from gel-filled baby teethers to a maximum ADI value of 10 mg kg^−1^ bodyweight [36] for girls and boys

Baby teether	Paraben amount (mean; µg 10 cm^−1^)	Percentage (%) of ADI^a^	
		Girls	Boys
No. 1	106	0.18	0.17
No. 2	149	0.26	0.23
No. 3	79	0.14	0.12
No. 4	148	0.25	0.23

Mean concentration value from products subjected to migration tests with water and saliva simulant (i.e. total n = 4) after a contact time of 1 h

^a^Calculated assuming bodyweights of 5.8 kg for girls and 6.4 kg for boys at the age of 3 months [37–39]

An observational study of object mouthing behaviour by young children examined the average daily mouthing duration for pacifiers, teethers, plastic toys, and other objects for children up to the age of 3 years in a normal environment (primarily home) [40]. In Phase I (pilot) and Phase II (including more participants) the children were divided into age groups of 0–18 months and 19–36 months, and observed for 1 day in both phases. In Phase III (final phase), observations were conducted for 5 nonconsecutive days over 2 months for children aged 3–18 months (at study initiation). Furthermore, the latter phase excluded pacifiers so that the observations were more focused on total mouthing time of nonpacifier objects, such as teethers, plastic toys, and other objects.

The obtained results showed that the mean mouthing duration of teethers for children aged 0–18 months was 6 min day^−1^ [40]. Taking this result and the total paraben amounts (mean detected amount in water and saliva simulant) released from each teether (Table 4) into account, the ADI value of 10 mg kg^−1^ using teethers nos. 1–4 would be reached after 3895–7333 days by girls and after 4298–8092 days by boys. In the 19–36 months age group, only one child out of every 110 used a teether, and the mean daily mouthing duration of all participants was considerably lower than 1 min. Therefore, exposure calculations were not performed for this age group in the present study. The mean mouthing duration of nonpacifier objects (the mean total mouthing duration of teethers, plastic toys, and other objects) is estimated to be 36 min day^–1^ for children aged 3–18 months (at study initiation) [40]. Based on this estimation, the ADI value of 10 mg kg^−1^ using teethers nos. 1–4 would be reached after 649–1222 days for girls and 716–1349 days for boys. Calculations based on mouthing duration from Phases I and II (pooled data) and Phase III were made based on bodyweights of 5.8 kg for girls and 6.4 kg for boys at the age of 3 months, as described above. According to this risk assessment, the ADI value would never be reached because the exposure time required surpasses the length of time that girls and boys usually use teethers.

However, specific research studies concerning different mouthing behaviours by infants and young children for items with cooling effects, such as gel-filled baby teethers in the present study, relating to conscious use due to the pain-relief effect would help achieve an adequate risk assessment to further estimate the exposure of infants and young children to parabens. Independent of this, a rough estimate of a worst-case scenario is possible using calculations based on detected paraben amounts released into water and saliva simulant. If continuous paraben release would be unchanged over time, the ADI value of 10 mg kg^−1^ using teethers nos. 1, 2, 3, and 4 with permanent mouthing would be reached after approximately 551, 390, 733, and 393 h by girls, and approximately 608, 430, 809 and 433 h by boys, respectively. However, the long-term migration study conducted to examine the continuous release behaviour of the parabens showed that the release level significantly decreased over time. Therefore, extrapolations based on results obtained in the 1-h migration tests can be considered overestimates.

### Influence of EVA polymer on paraben migration behaviour

Apart from different initially applied amounts of parabens during manufacturing, the main factor contributing to paraben release behaviour might be the VA content of copolymer EVA [41]. With increasing VA content, the crystallinity decreases and EVA becomes more elastic. Therefore, its permeability to gases, moisture, fats, and oils increases. Furthermore, with increasing molecular weight, the viscosity, toughness, heat seal strength, hot tack, and flexibility also increase. The main advantage of EVA over plasticised PVC is that no leachable plasticiser (such as phthalic acid ester) is needed, which makes EVA the material of choice for certain food applications, such as stretch film for food packaging, particularly for fresh meat. The influence of VA content on the diffusion behaviour of different organic compounds is already known. Furthermore, the diffusion properties of EVA membranes with varying VA content and in combination with the effect of vehicle ethanol concentration have been examined for benzocaine [42]. Results showed that, for any tested ethanol concentration, the solubility and diffusivity of benzocaine also increased with increasing VA content due to the amorphous nature of VA. Benzocaine is the ethyl ester of *p*-aminobenzoic acid and differs from EtP by only the amino group (–NH_2_), while the benzene ring of EtP has a hydroxyl group (–OH) substituted at the para position instead. In another study on benzoic acid, chlorobenzoic acid, methoxybenzoic acid, methylbenzoic acid, and nitrobenzoic acids, results also confirmed that the diffusion process was connected to the VA content of EVA membranes [43].

The demonstrated nonlinear decreasing trend in paraben release from the EVA-based chewing surface of baby teether no. 2 into water (see Fig. 4) was also based on the diffusion process of MeP and *n*-PrP between the EVA polymer and the gel-filled section, and the solubilities in both sections. It was assumed that the parabens were homogeneously distributed throughout the entire polymer section, and that a constant partition equilibrium was present between the polymer and gel-filled compartment at the beginning of the migration test. Their migration within the chewing section might be slower than their release from the region of the boundary layer into water (depending on the VA content). Therefore, the initially detected paraben amounts decreased with every water change until the paraben concentration reached an approximately constant concentration in the water phase. Different swelling effects on both sides of the EVA polymer might also be caused by the gel core and water, and would, consequently, have an additional effect on the transport of parabens into the chewing section and their release from the polymer surface into water. The degree of swelling of EVA polymer in pure water and ethanol–water mixtures has been examined in connection with the VA content [42].

Chen and Lostritto [42] determined the constant concentration transfer of benzocaine through EVA polymer using a side-by-side glass diffusion cell. The constant concentration flux of benzocaine into the EVA polymer was realised using a benzocaine saturated donor solution. The donor and receiver solutions consisted of the same matrix and volumes, and the receiver solution was changed at appropriate intervals. The results showed that the benzocaine amount crossing the EVA polymer with a membrane thickness of 0.051 mm and a VA content of 9% into water at 25 °C was approximately 40 µg cm^−2^ after 360 min [42]. Accordingly, for the membrane surface of 10 cm^2^ and 1-h contact time used in the present study, the benzocaine transferred would correspond to approximately 67 µg. This suggested that similar results could be obtained for parabens due to their similar molecular structures. Furthermore, a paraben-saturated EVA-based chewing section of approximately 1–2 mm might be comparable to an EVA membrane 0.051 mm in thickness and a saturated benzocaine solution. The results of the present study seem to confirm this assumption, with concentrations ranging from 38 to 99 µg 10 cm^−2^ h^−1^ and 30 to 99 µg 10 cm^−2^ h^−1^ (mean total paraben amount released into water and saliva simulant) for MeP and *n*-PrP, respectively. Therefore, the results of the benzocaine migration study [42] indicated that the paraben release behaviour from teethers might depend on the VA percentage of the polymer.

## Conclusions

Following a previous study that confirmed qualitatively that MeP, EtP, and *n*-PrP could leach from an EVA-based chewing surface of a gel-filled baby teether into water [9], the present study focused on establishing an accurate, reliable, and fast analytical method to quantify these EDCs. Furthermore, the migration tests applied provided representative in vitro migration data to estimate possible hazard presented by this source.

The SPE/GC–MS combined with SIDA method applied to analyse MeP, EtP, and *n*-PrP released from the EVA-based chewing surfaces of gel-filled baby teethers showed good validation results. Therefore, this analytical procedure is suitable for the analysis of parabens in water and saliva simulant migration test solutions.

The presented sample analyses confirmed the results and substantiated the findings of a previous study [10]. The present study also demonstrated that parabens in the polymer-based EVA chewing surface of the gel-filled baby teethers could be released into water or saliva simulant at approximately 50–150 µg 100 mL^−1^. Furthermore, a long-term migration study showed that the exposure of infants and young children to parabens by teethers can continue over an extended time period. However, under normal use behaviour, the total amount of parabens released over the entire use period would never reach the ADI for adults. Nevertheless, these findings are not negligible, because specific ADIs for children can be much lower than those for adults due to a possible higher sensitivity. The special caution shown towards children is exemplified by the prohibition of paraben preservatives as additives in food for infants and young children [21, 22].

Currently, there is no legal restriction on the use of parabens in gel-filled baby teethers. Nevertheless, manufacturers of gel-filled baby teethers should clarify why using preservatives, particularly parabens, is necessary. As microbial growth is highly conceivable in a gel due to the high water content, preservatives have probably been purposefully deployed in higher amounts than measured in the present and previous study [10]. If preservatives are required to avoid microbial growth, the use of polymer compositions with appropriate barrier properties towards preservatives such as parabens is required. Furthermore, four baby teethers contained sorbic acid in the gel, but not in the EVA polymer. Therefore, sorbic acid might be appropriate and applicable if gel preservation is needed. This might be more associated with the high solubility of its salts in water, which prevents diffusion from the watery gel to the organic plastic, than with the barrier properties of EVA. Although the quantitative findings of both studies are not alarming, it would be desirable for manufacturers of gel-filled baby teethers or similar products to avoid the use of such preservatives completely. However, two of the analysed gel-filled baby teethers did not contain detectable parabens, showing that not all manufacturers use these substances.

## Data Availability

Not applicable.

## Abbreviations

ADI: acceptable daily intake
BSTFA: *N*,*O*-bis(trimethylsilyl)trifluoroacetamide
EDCs: endocrine-disrupting compounds
EFSA: European Food Safety Authority
EI: electron ionisation
EtP: ethylparaben
EVA: ethylene-vinyl acetate
FTIR: Fourier transform infrared spectroscopy
GC–MS: gas chromatography–mass spectrometry
HCl: hydrochloric acid
LLE: liquid–liquid extraction
LOD: limit of detection
LOQ: limit of quantification
LRIs: linear retention indices
MeP: methylparaben
MeP-*d*_*4*_: methylparaben-*d*_*4*_
*n*-BuP: *n*-butylparaben
*n*-PrP: *n*-propylparaben
PVC: polyvinyl chloride
RSDs: relative standard deviations
SIDA: stable isotope dilution assay
SPE: solid-phase extraction
UAE: ultrasound-assisted extraction
VA: vinyl acetate

## References

[1] Rastogi SC (1998). Gas chromatographic analysis of phthalate esters in plastic toys. Chromatographia.

[2] Gill US, Lalonde PJ, Chantal PD, Subramanian KS (1999). Analysis of diisononyl phthalate in PVC consumer products used by children. Int J Consum Prod Saf.

[3] Earls AO, Axford IP, Braybrook JH (2003). Gas chromatography–mass spectrometry determination of the migration of phthalate plasticisers from polyvinyl chloride toys and childcare articles. J Chromatogr A.

[4] Al-Natsheh M, Alawi M, Fayyad M, Tarawneh I (2015). Simultaneous GC–MS determination of eight phthalates in total and migrated portions of plasticized polymeric toys and childcare articles. J Chromatogr B.

[5] Diamanti-Kandarakis E, Bourguignon JP, Giudice LC, Hauser R, Prins GS, Soto AM, Zoeller RT, Gore AC (2009). Endocrine-disrupting chemicals: an Endocrine Society scientific statement. Endocr Rev.

[6] Council of the European Communities (1976) Council Directive 76/769/EEC of 27 July 1976 on the approximation of the laws, regulations and administrative provisions of the Member States relating to restrictions on the marketing and use of certain dangerous substances and preparations, last amended 1 June 2009 and repealed by: Regulation (EC) No 1907/2006 of the European Parliament and of the Council of 18 December 2006. Official Journal of the European Union. L 262:201–203

[7] European Parliament and Council (2005) Directive 2005/84/EC of 14 December 2005 amending for the 22nd time Council Directive 76/769/EEC on the approximation of the laws, regulations and administrative provisions of the Member States relating to restrictions on the marketing and use of certain dangerous substances and preparations (phthalates in toys and childcare articles). Official Journal of the European Union. L 344:40–43

[8] European Parliament and Council (2006) Regulation (EC) No 1907/2006 of 18 December 2006 concerning the Registration, Evaluation, Authorisation and Restriction of Chemicals (REACH), establishing a European Chemicals Agency, amending Directive 1999/45/EC and repealing Council Regulation (EEC) No 793/93 and Commission Regulation (EC) No 1488/94 as well as Council Directive 76/769/EEC and Commission Directives 91/155/EEC, 93/67/EEC, 93/105/EC and 2000/21/EC, last amended 13 June 2017. Official Journal of the European Union. L 396:1–849

[9] Berger E, Potouridis T, Haeger A, Püttmann W, Wagner M (2015). Effect-directed identification of endocrine disruptors in plastic baby teethers. J Appl Toxicol.

[10] Potouridis T, Berger E, Püttmann W (2016). Analysis of alkyl esters of *p*-hydroxybenzoic acid (parabens) in baby teethers via gas chromatography–quadrupole mass spectrometry (GC–qMS) using a stable isotope dilution assay (SIDA). Anal Methods.

[11] Routledge EJ, Parker J, Odum J, Ashby J, Sumpter JP (1998). Some alkyl hydroxy benzoate preservatives (parabens) are estrogenic. Toxicol Appl Pharmacol.

[12] Byford JR, Shaw LE, Drew MGB, Pope GS, Sauer MJ, Darbre PD (2002). Oestrogenic activity of parabens in MCF7 human breast cancer cells. J Steroid Biochem Mol Biol.

[13] Boberg J, Taxvig C, Christiansen S, Hass U (2010). Possible endocrine disrupting effects of parabens and their metabolites. Reprod Toxicol.

[14] Soni MG, Carabin IG, Burdock GA (2005). Safety assessment of esters of *p*-hydroxybenzoic acid (parabens). Food Chem Toxicol.

[15] Stopforth JD, Sofos JN, Busta FF, Davidson PM, Sofos JN, Branen AL (2005). Sorbic acid and sorbates. Antimicrobials in food.

[16] Peck AM (2006). Analytical methods for the determination of persistent ingredients of personal care products in environmental matrices. Anal Bioanal Chem.

[17] Sabalitschka T (1930). Verwendung der p = Oxybenzoesäureester zur Sterilhaltung, Sterilisation und Desinfektion. Arch Pharm.

[18] Eisenbrand G, Schreier P (2006). RÖMPP Lexikon der Lebensmittelchemie.

[19] European Parliament and Council (2009) Regulation (EC) No 1223/2009 of 30 November 2009 on cosmetic products, last amended 3 August 2017. Official Journal of the European Union. L 342:59–209

[20] European Parliament and Council (2014) Commission Regulation (EU) No 1004/2014 of 18 September 2014 amending Annex V to Regulation (EC) No 1223/2009 of the European Parliament and of the Council on cosmetic products. Official Journal of the European Union. L 282:5–8

[21] European Parliament and Council (2008) Regulation (EC) No 1333/2008 of 16 December 2008 on food additives, last amended 28 July 2017. Official Journal of the European Union. L 354:16-33

[22] European Parliament and Council (2011) Commission Regulation (EU) No 1129/2011 of 11 November 2001 amending Annex II to Regulation (EC) No 1333/2008 of the European Parliament and of the Council by establishing a Union list of food additives. Official Journal of the European Union. L 295:1–177

[23] Könemann WH. Phthalate release from soft PVC baby toys. Report from the Dutch Consensus Group, RIVM report 613320 002. National Institute for Public Health and the Environment, Dutch Ministry of Health, Welfare and Sport, Bilthoven; 1998

[24] Milo C, Blank I (1998). Quantification of impact odorants in food by isotope dilution assay: strength and limitations. ACS Symp Ser.

[25] Rychlik M, Asam S (2008). Stable isotope dilution assays in mycotoxin analysis. Anal Bioanal Chem.

[26] Rychlik M, Asam S (2009). Stable isotope dilution assays for quantitation of organic trace compounds in food analysis. Umweltwiss Schadst Forsch.

[27] Azzouz A, Ballesteros E (2014). Trace analysis of endocrine disrupting compounds in environmental water samples by use of solid-phase extraction and gas chromatography with mass spectrometry detection. J Chromatogr A.

[28] van den Dool H, Kratz PD (1963). A generalization of the retention index system including linear temperature programmed gas–liquid partition chromatography. J Chromatogr.

[29] Zellner BD, Bicchi C, Dugo P, Rubiolo P, Dugo G, Mondello L (2008). Linear retention indices in gas chromatographic analysis: a review. Flavour Fragr J.

[30] Deutsches Institut für Normung (2008). DIN 32645. Chemical analysis—decision limit, detection limit and determination limit under repeatability conditions—terms, methods, evaluation.

[31] von Soxhlet F (1879). Die gewichtsanalytische Bestimmung des Milchfettes. Dingler’s Polytechnisches J.

[32] Poole SK, Dean TA, Oudsema JW, Poole CF (1990). Sample preparation for chromatographic separations: an overview. Anal Chim Acta.

[33] Kou D, Mitra S, Mitra S (2003). Extraction of semivolatile organic compounds from solid matrices. Sample preparation techniques in analytical chemistry.

[34] Kapalavavi B, Ankney J, Baucom M, Yang Y (2014). Solubility of parabens in subcritical water. J Chem Eng Data.

[35] Asimakopoulos AG, Elangovan M, Kannan K (2016). Migration of parabens, bisphenols, benzophenone-type UV filters, triclosan, and triclocarban from teethers and its implications for infant exposure. Environ Sci Technol.

[36] European Food Safety Authority (2004). Opinion of the Scientific Panel on food additives, flavourings, processing aids and materials in contact with food on a request from the Commission related to para hydroxybenzoates (E 214-219). EFSA J.

[37] de Onis M, Martorell R, Garza C, Lartey A (2006). WHO child growth standards based on length/height, weight and age. Acta Paediatr.

[38] WHO Multicentre Growth Reference Study Group. WHO Child Growth Standards: Length/height-for-age, weight-for-age, weight-for-length, weight-for-height and body mass index-for-age: methods and development. Geneva: World Health Organization; 2006. http://www.who.int/childgrowth/standards/en/. Accessed 29 Mai 2015

[39] Centers for Disease Control and Prevention (CDC). Growth charts. Atlanta (GA): CDC 2010. http://www.cdc.gov/growthcharts/who_charts.htm. Accessed 29 May 2015

[40] Juberg DR, Alfano K, Coughlin RJ, Thompson KM (2001). An observational study of object mouthing behavior by young children. Pediatrics.

[41] Robertson GL (2013). Food packaging: principles and practice.

[42] Chen SX, Lostritto RT (1996). Diffusion of benzocaine in poly(ethylene-vinyl acetate) membranes: effects of vehicle ethanol concentration and membrane vinyl acetate content. J Control Release.

[43] Maurin MB, Dittert LW, Hussain AA (1992). Mechanism of diffusion of monosubstituted benzoic acids through ethylene-vinyl acetate copolymers. J Pharm Sci.

